# Medicine

**Published:** 1891-01

**Authors:** DeLancey Rochester


### MEDICINE.

CONDUCTED BY

DELANCEY ROCHESTER, M. D.

### THE CONDITION OF THE HEART IN ANEMIA, AND THE CAUSE OF THE PULMONARY MURMUR.

HANDFORD (*American Journal of the Medical Sciences*, Vol. C., No. 6, p. 558,) presents an original and most interesting view of the cause of the pulmonary murmur in anemia. He agrees with all other careful observers, that in anemia the heart muscle undergoes some fatty degeneration and the cavities become dilated. He considers that this dilatation is greater on the right side, and gives good reasons for his conclusions. He brings forward the view, which is undoubtedly correct, that "there are several different murmurs in anemia, one or all, or none, of which may be audible in a given case."

He states that "the characteristic of the so-called hemic, pulmonary murmur is that, though audible frequently in all the areas, its point of maximum intensity is in the second or third left interspace (usually the second), close to the sternum ; and that it invariably becomes much less loud, and frequently totally disappears in the erect position." He cites several cases in support of this view.

His theory of the cause of the pulmonary murmur, in support of which this article is written, is that "this pulmonary murmur, disappearing in the erect position, is due to the pressure of an enlarged, flabby, and dilated heart on the pulmonary artery."

It is one of the most interesting papers that has been written, and shows careful observation and deep study of cases.

---

### GASTRO-INTESTINAL DISORDERS AND THE DIET IN PULMONARY PHTHISIS.

KARL VON RUCK (*Therapeutic Gazette*, Vol. XIV., No. 10,) gives the result of some observations on this subject. He brings forward the statement that in many cases of phthisis, the first — and for a long time the only — indication of tubercular trouble is the continuous and obstinate existence of indigestion. This is, of course, an old idea, but the detailed account of the management of cases shows how much can be done for their improvement by a conscientious worker. The ideas thrown out are overfeeding, carefully and scientifically done, the hydrocarbons being considerably in excess, rest in bed with massage for exercise, gastric lavage when indicated, and, in the way of medication, the use of the pneumatic cabinet, and oxygen, together with creosote in rapidly increasing doses, and dilute hydrochloric acid, when that factor is diminished in or absent from the gastric juice.

